# Productivity-Diversity Relationships in Lake Plankton Communities

**DOI:** 10.1371/journal.pone.0022041

**Published:** 2011-08-05

**Authors:** Jenni J. Korhonen, Jianjun Wang, Janne Soininen

**Affiliations:** 1 Department of Environmental Sciences, University of Helsinki, Helsinki, Finland; 2 State Key Laboratory of Lake Science and Environment, Nanjing Institute of Geography and Limnology, Chinese Academy of Sciences (CAS), Nanjing, China; University of Aberdeen, United Kingdom

## Abstract

One of the most intriguing environmental gradients connected with variation in diversity is ecosystem productivity. The role of diversity in ecosystems is pivotal, because species richness can be both a cause and a consequence of primary production. However, the mechanisms behind the varying productivity-diversity relationships (PDR) remain poorly understood. Moreover, large-scale studies on PDR across taxa are urgently needed. Here, we examined the relationships between resource supply and phyto-, bacterio-, and zooplankton richness in 100 small boreal lakes. We studied the PDR locally within the drainage systems and regionally across the systems. Second, we studied the relationships between resource availability, species richness, biomass and resource ratio (N∶P) in phytoplankton communities using Structural Equation Modeling (SEM) for testing the multivariate hypothesis of PDR. At the local scale, the PDR showed variable patterns ranging from positive linear and unimodal to negative linear relationships for all planktonic groups. At the regional scale, PDRs were significantly linear and positive for phyto- and zooplankton. Phytoplankton richness and the amount of chlorophyll *a* showed a positive linear relationship indicating that communities consisting of higher number of species were able to produce higher levels of biomass. According to the SEM, phytoplankton biomass was largely related to resource availability, yet there was a pathway via community richness. Finally, we found that species richness at all trophic levels was correlated with several environmental factors, and was also related to richness at the other trophic levels. This study showed that the PDRs in freshwaters show scale-dependency. We also documented that the PDR complies with the multivariate model showing that plant biomass is not mirroring merely the resource availability, but is also influenced by richness. This highlights the need for conserving diversity in order to maintain ecosystem processes in freshwaters.

## Introduction

In recent decades, the number of studies examining the factors affecting species richness in ecosystems has greatly increased. This increase results partly from the ongoing global decline in biodiversity caused by humans. In recent years, studies have especially addressed the causes of diversity patterns along specific gradients such as altitude [1] and latitude [2]. Moreover, the current recognition of the pivotal role of diversity in ecosystem functioning and services has enhanced the interest in studies on biodiversity [3].

**Figure 1:**
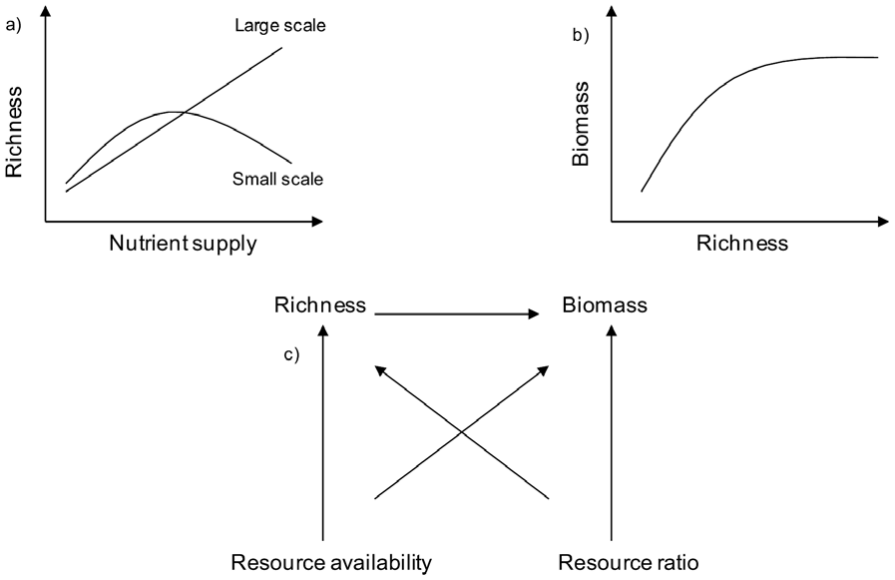
A conceptual figure of the hypotheses. a) At small and large scale, the relationships between species richness and nutrient supply are predicted to be unimodal or linear, respectively. b) Biomass production first increases with species richness but saturates at high richness levels. c) The causal relationships between resource availability, species richness, biomass and resource ratio. Figure modified from Cardinale et al (8).

One of the most interesting gradients associated with the variation in species diversity is ecosystem productivity. Given the predominant role of productivity for species coexistence, the relationship between productivity and diversity (PDR) has become a fundamental research area in modern ecology (e.g. [4]). The relationship has direct applications for many central environmental issues, such as biodiversity conservation and ecosystem functions and services. The role of diversity in ecosystems is remarkable, because species richness can be both a cause and a consequence of primary production, i.e. the rate of carbon fixed through photosynthesis [5], [6]. This dual role of biodiversity is based on two theories. First, the species-energy theory suggests that the amount of resource supply determines the number of coexisting species (Fig. 1, [7]). Second, the studies in the field of biodiversity-ecosystem functioning (BEF) are built on the premise that the species richness controls the biomass production of a community (Fig. 1, [8]). Combined with the resource ratio theory [9], these theories have also led to formulation of the multivariate hypothesis of PDR [8].

Even though the PDR has been widely examined using experimental approaches and observations, the underlying mechanisms still remain poorly understood. However, one of the most common mechanisms behind the positive PDR is the *sampling effect*. This mechanism is based on the assumption that more diverse communities are more likely to include species that are especially effective in capturing resources and converting these into plant biomass [10]–[12]. The sampling effect is expected to affect ecosystem functioning especially in the studies spanning short temporal extents (reviewed in [13]) or in studies conducted in homogenous environments [14] or the landscape [15]. Another mechanism driving the PDR is *complementarity*, i.e. niche differentiation between the species present in a community. A positive complementarity effect represents the sum of all biological processes involving two or more species, positively influencing a focal process, such as niche partitioning and facilitation [16]. This effect is based on a view that more diverse communities function more efficiently, because ecologically different species that compete for limiting resources are present and species thus complement each other in their resource use [10], [11]. Niche complementarity is expected to affect productivity in the long-term only as species' differences in resource use typically needs enough time to have functional consequences in the ecosystems [13].

Both sampling effect and complementarity may cause positive linear PDR, and this type of relationship is, indeed, common in nature. However, reviews suggest that unimodal relationships are also typical especially in plant communities and in aquatic ecosystems [4], [17]. In unimodal relationships, the number of species peaks at intermediate productivity. The low number of species at low and high ends of productivity gradient can result from small amount of resources and intense competition, respectively [4]. Moreover, positive interspecific interactions (i.e., *facilitation*) can explain the coexistence of large number of species at the intermediate productivity [18].

Besides being affected by biological processes, the shape of the PDR is likely to be driven by the spatial scale of the study. In aquatic ecosystems, unimodal PDR are more common in studies covering small (local) scales, while positive linear relationships tend to dominate in studies covering larger (regional) scales [19], [20]. The main reason for the scale-dependency is the increase of species dissimilarity with productivity within regions, i.e., more productive lakes or streams have more *multiple stable states* [20], [21]. The generality of this scale-dependency in PDR across organisms has, however, remained unresolved, as studies testing the scale-dependency are usually conducted in disparate systems using different methods. The cross-taxonomic group comparisons of PDR are, however, important given that PDR can be mediated by different mechanisms across organism groups that vary in body size, trophic position [22] or dispersal capacity [23]. Some pioneer studies on PDR in phytoplankton communities have been conducted (e.g. [20], [24]), but more large-scale studies on PDR across taxa in natural unmanipulated ecosystems are urgently needed. We emphasize also that the PDRs are largely understudied for small organisms such as lake bacteria (but see [25], [26]). Bacteria are interesting not only due to their small size and efficient dispersal, but also because they have a unique functional role representing decomposers in nature.

In this study, we first (i) examine the relationships between resource supply and richness of bacterio-, phyto-, and zooplankton in 100 small lakes in Finland. We expect that the patterns in PDR between micro- (bacterio- and phytoplankton) and macroorganisms (zooplankton) may well differ because, being small and often highly abundant, microorganisms may show virtually unrestricted dispersal [27]. According to Pärtel & Zobel [23], species that show dispersal limitation are likely to show unimodal PDRs if species pool size and the degree of biotic interactions do not vary along productivity while patterns are more likely to be linear for highly dispersive taxa. We examine the PDR at two different scales: (A) locally among 20 lakes sampled within each drainage system, and (B) regionally among the five sampled drainage systems, i.e., across all 100 lakes that were sampled. We thus vary the extent of the study from one to five drainage systems but keep the focus of research the same (one lake). Second (ii), we examine the relationship between phytoplankton species richness and standing biomass and expect that biomass increases with species richness because of enhanced ecosystem functioning (Fig. 1, [8]). Here, we also relate phytoplankton community composition with biomass to see if species composition is related to biomass, suggesting that the productivity is also affected by composition effects [28]. Finally (iii), we test the multivariate hypothesis of PDR suggested by Cardinale et al [8] and study the relationships between resource availability, species richness, biomass and resource ratio (N∶P) in phytoplankton communities using Structural Equation Modeling (see [8]). Following Cardinale et al [8], we expect that phytoplankton biomass in lakes is largely determined by resource availability, yet is also driven by phytoplankton richness and resource ratio (Fig. 1).

## Materials and Methods

### Study area

Bacterio-, phyto-, and zooplankton were collected once from 100 small lakes in Finland during July in 2008 and 2009. In case of residential areas (summer cottages), we asked oral permissions from the land owners to take water samples from the nearby lakes. However, most of the study lakes were several kilometers away from the nearest settlements. Therefore, the everyman's right of Finland allowed us to access the lands and lakes as we did not fish, harm or disturb the natural environment.

The sites were sampled at five drainage systems, 20 lakes per system. In 2008, we sampled 60 lakes at three drainage systems and in 2009 40 lakes at two drainage systems. We acknowledge that between-year variation in environmental conditions may increase the residual variation in the data that could not be controlled. However, sampling of 100 lakes during a single summer was not possible due to seasonality and substantial increase of within-year variation in the data. We also acknowledge that a single sampling may not always accurately reflect the true number of species occupying lakes. However, according to Shurin et al [29], daily richness and annual richness were highly correlated for zooplankton in 36 lakes in a temperate region. We thus think that our sampling design represented among-lake differences in richness relatively well.

**Figure 2:**
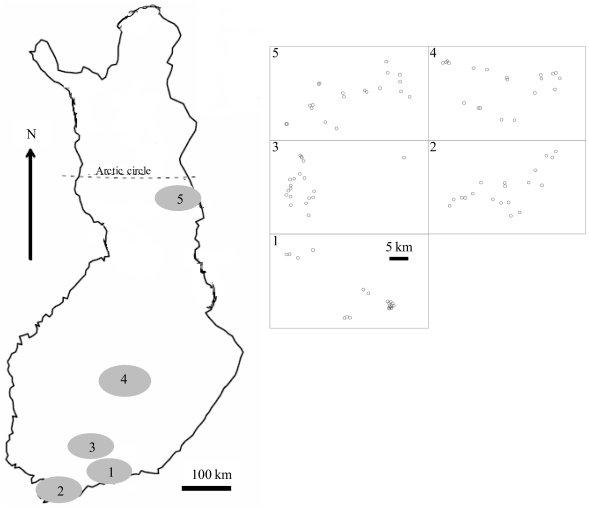
Map of Finland with the study areas marked by gray circles. The study areas were: 1) Vantaanjoki, 2) Karjaanjoki, 3) Kokemäenjoki, 4) Upper Kymijoki, and 5) Koutajoki. On the right, small maps show geographical positions of each lake in the same study areas.

The sampled drainage systems were (1) Vantaanjoki, (2) Karjaanjoki, (3) Kokemäenjoki, (4) Upper Kymijoki, and (5) Koutajoki (Fig. 2). These drainage systems were chosen, because they cover a large geographical extent and their nutrient concentrations vary from ultraoligotrophic to highly eutrophic. Latitudinal gradient between the southernmost and the northernmost sites was more than 700 km. We sampled only small lakes and ponds to ensure that plankton sampling covered the site as well as possible. Most of the lakes within the drainage systems were not readily inter-connected to each other via water routes. For more information on the environmental characteristics of the lakes within the drainage systems, please see Table S1.

### Sampling and sample processing

Plankton samples were collected from the middle of each lake using a tube sampler (V = 2.3 L) at three locations, which were pooled.We collected the samples in the middle of the lakes in order to avoid benthic taxa from the littoral entering the samples. The samples were collected at 0.5 m below the surface of the water. Our sampling protocol for bacteria followed the method by Longmuir et al [30]. First 250 mL of water was filtered through a 0.42 µm pore-sized nitrocellulose filter (diameter 25 mm, Millipore, Durapore®) to remove larger particles. Bacteria cells were then collected on a 0.22 μm pore-sized nitrocellulose filter, which was frozen immediately in the field. Phytoplankton subsamples were mixed, and a sample of 0.5 L was fixed immediately with acid Lugol's iodine solution in the field. Zooplankton samples (6.15 L in total) were filtered through a 50 μm net and preserved with formaldehyde in the field.

The maximum depth of the lakes as well as surface water temperature was measured. We included surface water temperature as an explaining variable in the data because it showed notable differences among the sampled drainage systems (Table S1). Area of each lake was measured using Geographic Information System. Samples for water chemistry analyses were collected simultaneously with the plankton sampling and analyzed in the laboratory for conductivity, chlorophyll a (Chl a), water colour, total nitrogen, and total phosphorus using national standards. Water colour was determined using a comparator and nutrients using Lachat Quik-Chem 8000.

In the laboratory, the phytoplankton samples were concentrated using an Utermöhl chamber and counted with a light microscope (magnification 400×). For each sample, 50 fields were counted typically detecting 200–500 specimens (individuals or colonies). For zooplankton, all individuals (typically 50–200 individuals per sample) were counted at magnification of 125–400× using an inverted microscope. Both crustacean zooplankton and rotifers were included in countings. We acknowledge that our methodology for zooplankton does not detect as many individuals as it detects for bacteria or phytoplankton because of relatively limited amount of water filtered for the samples. However, as there was great among-lake variability in zooplankton richness, we feel that this methodology is adequate for inter-lake comparisons for zooplankton richness. For phyto- and zooplankton, most individuals were identified to species level. However, some of the taxa (<20%) were identified to genus level only. We thus acknowledge that within the data sets, not all taxa represent a species but rather a genus or even higher taxonomic groups for bacteria. This may mask some structure in the data only observable if species level data were used.

### Nucleic acid extraction and polymerase chain reaction (PCR)

For examining the community composition of bacteria, we used standard fingerprinting methods (see details below). Nitrocellulose filters were cut in half and placed into a 1.5 mL microtube which was then dipped in liquid nitrogen. The filters were then roughly ground with a plastic pestle and deoxiribonucleic acid (DNA) was extracted with a protocol of Griffiths et al [31] with the following modifications: 0.6 mL of extraction buffer and zirconium beads (Qiagen) were added to the ground filters in 2 mL tubes and mixed by vortexing. Once all the samples contained the extraction buffer, 0.6 mL of buffered (pH 8) Phenol∶Chloroform∶Isoamyl alcohol was added to each tube and vortexed again. Mechanical lysis was performed on a bead-beating device for 120 seconds at maximum speed (1800 rounds per minute). DNA was finally resuspended in 20 μL Tris Ethylenediaminetetraacetic (EDTA) acid buffer (10 mmol^−L^ Tris 1 mmol^−1^ EDTA).

As a molecular fingerprinting method, we used terminal restriction fragment length polymorphism (tRFLP) analysis [32]. It is a popular method for generating a fingerprint of an unknown microbial community. Although it may underestimate the true number of bacteria taxa present, our consistent methods allow us to investigate the distribution patterns of bacteria among the lakes. For the tRFLP analysis, PCR amplification of 16S ribosomal genes for tRFLP was achieved by using primers FAM-E8F (FAM-5′-AGAGTTTGATCCTGGCTCAG-3′) and E939R (5′-CTTGTGCGGGCCCCCGTCAATTC-3′) [33] with reaction conditions optimized for the enzyme DyNAzyme II (Finnzymes). PCRs were run in triplicate reactions, aliquots were checked by agarose gel electrophoresis separately and the rest of the volume was pooled. The pools were purified with a Millipore Multiscreen plate. The clean PCR products were digested with 5 units of restriction enzyme (HhaI, Fermentas) for 18 hours in duplicate reactions. Dilutions of the digested and undigested samples were run on an Applied Biosystems (ABI) 3130xl device at 60°C. The resulting peak profiles (taxonomic units) were analyzed using the ABI PeakScanner software. All peaks with a size of 50–940 base pairs (bp) and a relative height of at least 0.1% above the baseline present in both digestions were manually recorded for each sample and compared to profiles from undigested PCR products. The peaks that located closer than 2 bp from each other were binned. We used the limit of 2 bp for all fragment sizes.

### Statistical analyses

The degree of saturation in local communities was assessed using species-accumulation curves across sampled sites in each drainage system. We used the freely available software package Ecosim 7.0. (http://garyentsminger.com/ecosim.htm). The procedure was done to ensure that 20 sampled lakes covered the regional species pool sufficiently, e.g. included more than 70% of the species. Our data showed that a sample of 20 sites per region is likely to be adequate, as the curves seem steadily approach the asymptotes ().

The relationship between species richness and nutrient supply was analyzed using linear and quadratic regression with AIC (Akaike's Information Criterion) to select the best model. The relationships were analyzed at two spatial scales: within and across the drainage systems.

**Figure 3:**
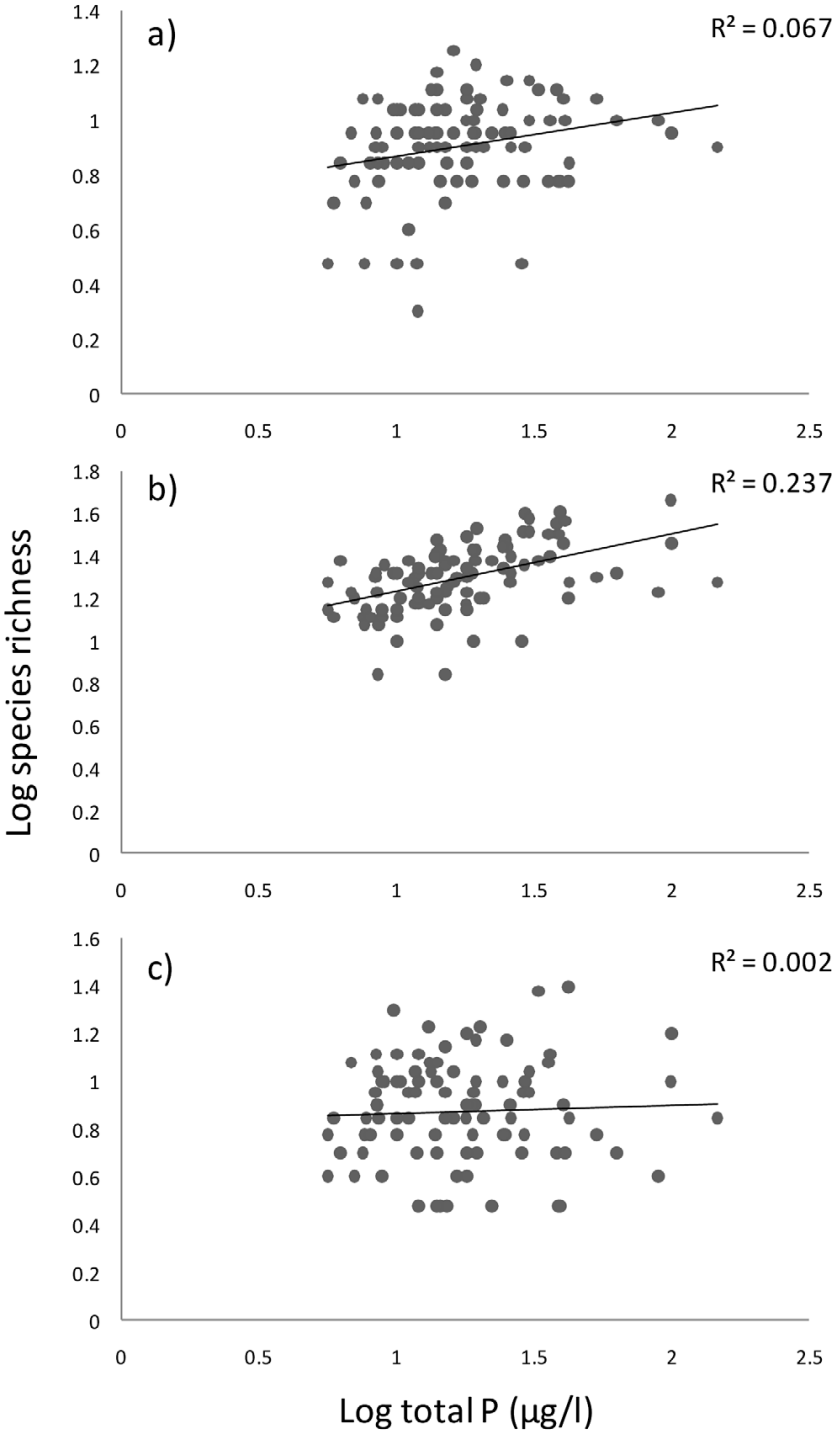
The relationships between log-transformed species richness and log total P (µg/l) in a) zooplankton, b) phytoplankton, and c) bacterioplankton data sets (n = 100).

Moreover, we used regression analysis to test the relationship between phytoplankton species richness and biomass. Analyses were done using SPSS 15.0 (SPSS, Inc.). Besides using observed richness values, we conducted analyses with richness values modified using Chao1 formula [34], [35], which should be useful for small organisms with highly skewed rank frequency distributions. However, the results were qualitatively highly similar with the observed species richness data (results not shown). Therefore, we used observed species richness as an indicator for diversity. All analyses were conducted using both non-transformed richness values and log-transformed values. As the main patterns were qualitatively similar, we show here the results for non-transformed richness values (except in Fig. 3). We also studied if phytoplankton community composition was related to phytoplankton biomass. This was done by regressing site NMDS (Non-Metric Multidimensional Scaling, [36]) 1 scores against phytoplankton biomass of a site. NMDS analysis was conducted using presence-absence data of the phytoplankton species with the R package 2.8. (www.r-project.org).

The relationships between resource ratio (N∶P), resource availability, species richness and phytoplankton biomass were examined using Structural Equation Modeling (SEM, [37]). We used SEM analysis only for the phytoplankton data as we did not have biomass measures for bacteria or zooplankton. SEM is an extension of GLM (General Linear Model) in which a set of regressions is solved simultaneously to examine whether a covariance matrix complies with a set of causal pathways set *a priori*. Total N and total P values were standardized to have a mean of zero and standard deviation of 1. The resource availability (a) and resource ratios (θ) were calculated using resource vectors from the two resource values (total N and total P) according to equations 2 and 4 in Cardinale et al [8]. This was done to separate the resource availability from resource imbalance between N and P. Resource ratio (θ) ranges from 0–90 with 0 meaning perfect balance, and 90 perfect imbalance, relative to the total variation among the sampled lakes. The goodness of fit of the full model was tested using Chi-square test. Chi-square with non-significance test indicates that there is no deviation between the observed covariance matrix and that predicted by SEM. Akaike's Information Criterion (AIC) was used to select the most parsimonious model. Using AIC, the final model was chosen based on the likelihood (AIC_L_) that the model was the best fit to current data set among the candidate models. We also conducted a full path model without model selection to show all related individual pathways. SEM was conducted in Amos 18.0 (SPSS, Inc.).

Finally, we studied which environmental, geographical or biological factors were strongest determinants of species richness for each planktonic group. We calculated the relationship between species richness and water chemistry (total P, total N, color, conductivity), water temperature, surface area, maximum depth and geographical location (latitude and longitude) of the lake using GLM with the best model selection by AIC. As the PDR is frequently unimodal, we also included the second order terms of total P and total N in the candidate models. The cross-taxon concordance between zooplankton, phytoplankton and bacterioplankton richness was analyzed including richness values into GLM models as well as with the separate correlation analyses. Analyses were conducted using R package 2.8. (www.r-project.org).

## Results

At within-drainage system scale, the PDR showed highly variable patterns in all organism groups ranging from positive linear and unimodal relationships with total P to negative linear relationships in some of the drainage systems. In zooplankton, the PDR was significantly unimodal only in the Koutajoki drainage system (Table 1, Figure S2). In the four other drainage systems, the PDR varied from positive linear to slightly negative linear but none of the relationships was significant (Table 1). In phytoplankton, two out of five drainage systems (Vantaanjoki and Upper Kymijoki) showed a significant PDR with positive linear and unimodal relationships, respectively (Table 1, Figure S2). Bacterioplankton richness and total P were unimodally related only in the Karjaanjoki drainage system (Table 1). All other relationships were non-significant.

**Table 1:** The regression models for the relationships between local species richness and concentrations of total P (µg/l) at five drainage systems and for the whole set of lakes for each planktonic group.

	Zooplankton			Phytoplankton			Bacterioplankton		
	*Model*	*R^2^*	*p*	*Model*	*R^2^*	*p*	*Model*	*R^2^*	*p*
Vantaanjoki	Linear	0.051	0.513	**Linear**	**0.433**	**0.002**	Linear	0.032	0.606
Karjaanjoki	Linear	0.058	0.332	Linear	0.170	0.154	**Unimodal**	**0.317**	**0.039**
Kokemäenjoki	Linear	0.041	0.402	Unimodal	0.244	0.093	Linear	0.074	0.716
Upper Kymijoki	Linear	0.019	0.578	**Unimodal**	**0.528**	**0.002**	Linear	0.032	0.689
Koutajoki	**Unimodal**	**0.342**	**0.028**	Linear	0.131	0.093	Unimodal	0.197	0.156
All regions	**Linear**	**0.0672**	**0.009**	**Linear**	**0.237**	**0.001**	Linear	0.0019	0.756

Linear or quadratic model is given depending on the AIC value of the model. The regression model for zooplankton in Karjaanjoki was negative linear as well as the model for bacterioplankton in Upper Kymijoki. The rest of the linear models were all positively correlated. Significant results are in bold. * Data for all regions were log-transformed.

Across regions comprising all 100 lakes that were sampled, there were significant linear relationships between log-transformed phytoplankton and zooplankton species richness and total P (R^2^ = 0.237; P = 0.001, R^2^ = 0.067, P = 0.009, respectively; Fig. 3a, b). Bacterioplankton richness did not show significant relationship with total P (R^2^ = 0.002 for the linear model ; P = n.s.; Fig. 3c). Relationships were slightly weaker, yet significant, for phyto- and zooplankton when non-transformed data were used (results not shown). Given that we found linear relationships across drainage systems covering the larger study scale, but variable patterns within the drainage systems, these results give overall partial support for the scale-dependency of the PDR in our study system.

**Figure 4:**
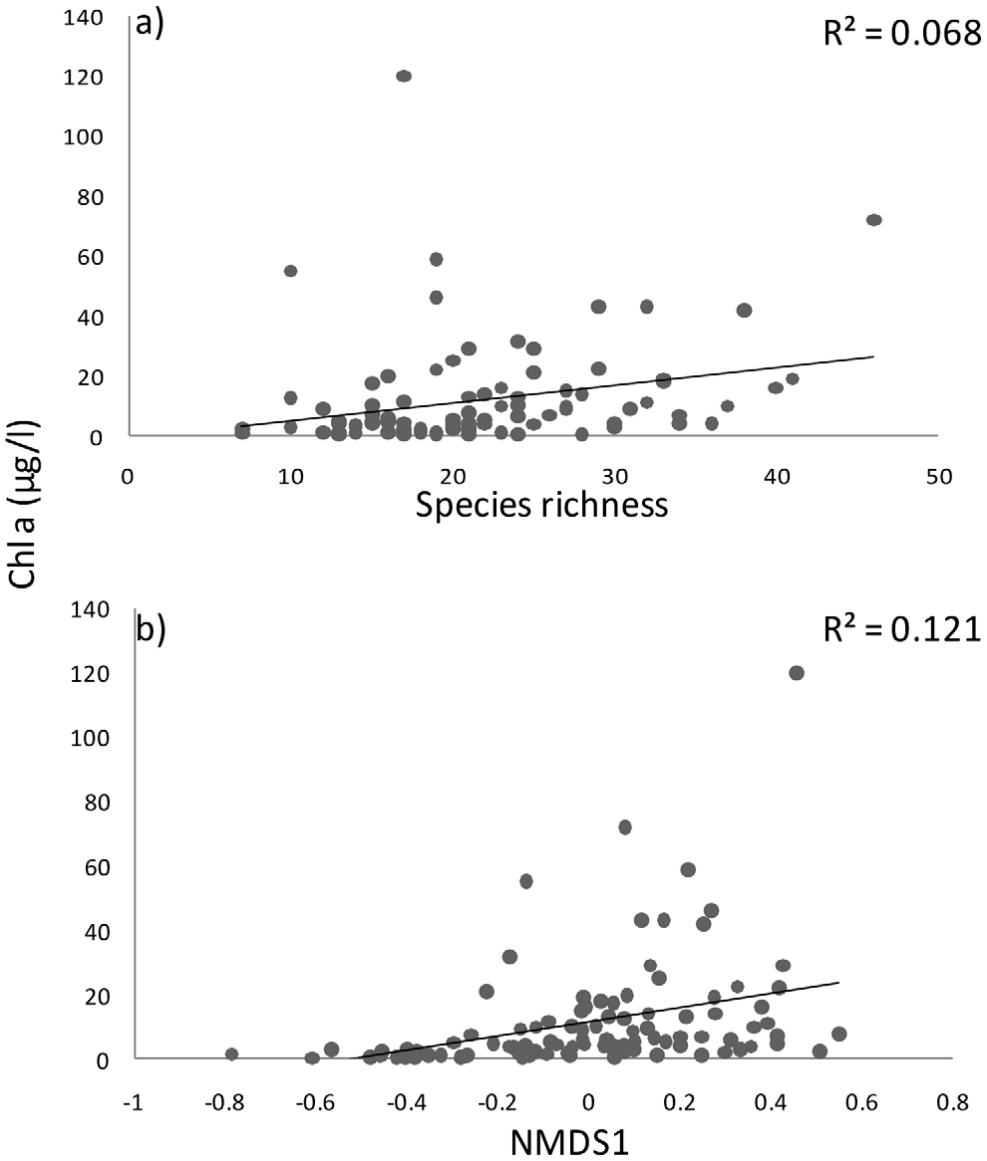
The relationship between a) phytoplankton richness (n = 100), and b) NMDS1 site scores and chl *a* (P = 0.009, and P<0.001, respectively).

Phytoplankton richness and the amount of chlorophyll *a* (µg/l) showed a positive linear relationship across the whole set of lakes (R^2^ = 0.0.068, P = 0.009; Fig. 4). This may indicate that the communities consisting of higher number of species were able to produce higher levels of biomass from basal resources. However, we acknowledge that the relationship can also be caused by the increasing number of species (and chlorophyll a), thus leading to a more species rich community. It also seems that community composition has either direct or indirect effects on standing biomass, as community composition (summarized by NMDS 1 scores) was related to phytoplankton biomass (R^2^ = 0.121, P<0.001; Fig. 4).

**Figure 5:**
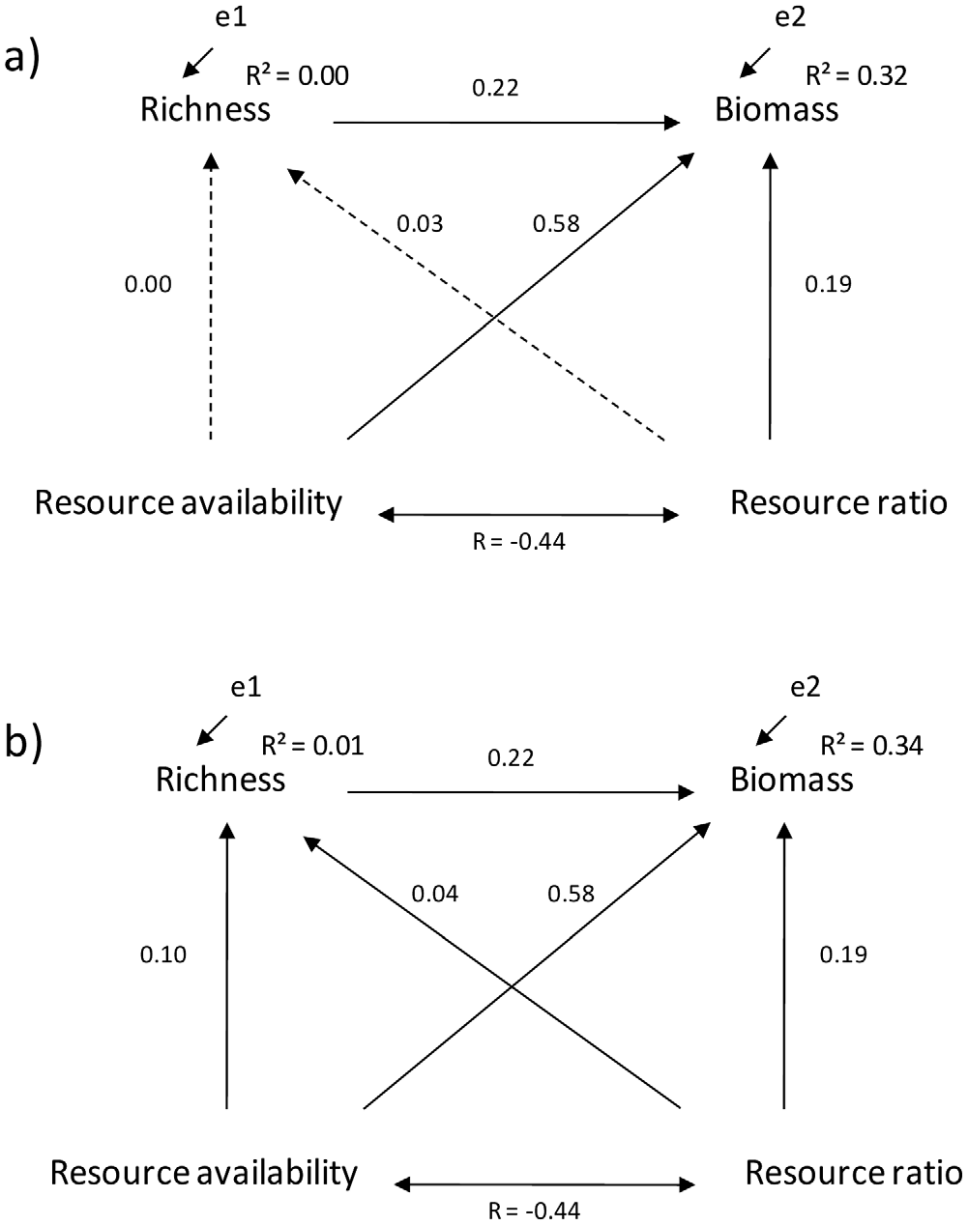
The results of a Structural Equation Modeling for phytoplankton data with (5a) or without (5b) best model selection. SEM was conducted to test whether covariance among variables collected from 100 lakes could be produced by a covariance matrix set *a priori* (shown in Fig. 1c). The coefficients next to arrows represent the standard deviation change between variables. R^2^ values indicate the amount of explained variation in species richness and phytoplankton biomass. The correlation between the resource supply and resource ratio (R) was −0.44. Dashed lines denote non-significant relationships.

In the SEM analysis for phytoplankton data, the assumptions concerning linear relationships were fulfilled (linearity of relationships, one-way causal flow, and variables measured on an interval or ratio scale). The chi-squared test indicated that there was no significant deviation between the observed covariance matrix and that predicted by the proposed SEM (Chi-square = 0.725, df = 2, P = n.s.). According to the best SEM model identified by AIC, phytoplankton biomass was largely related to resource availability (coefficient = 0.58), yet there was also a pathway via community richness (coefficient = 0.22) (Table S2, Fig. 5a). Surprisingly, there were no significant effects of resource availability on richness and resource ratio on richness in this model. However, full path model without model selection (Fig. 5b) showed a significant effect of resource availability on richness (coefficient = 0.10). In both models, the correlation between the resource supply and resource ratio was −0.44. Resource ratio and phytoplankton biomass were positively related. Overall, the best multivariate model explained 32% of the variation in phytoplankton biomass.

As planktonic richness was not only determined by ecosystem productivity, we studied whether it was related to some other physicochemical factors, location of the lake or richness of the trophic levels other than the focal planktonic group. The most parsimonious model for the whole zooplankton data included three variables (water temperature, bacterioplankton and phytoplankton richness), which were all positively correlated with the zooplankton richness (Table 2). The three variables jointly explained 21% of the variability in zooplankton richness. For the phytoplankton data set, the best model included five factors (Table 2). Electrical conductivity, longitude, total N, and zooplankton richness showed positive relationships with phytoplankton richness, while latitude and phytoplankton richness were negatively correlated (Table 2). The five variables jointly explained 48% of the variation in phytoplankton richness. Variation in bacterioplankton richness, in turn, was mainly related to geographical position of the lake and zooplankton richness. Longitude was negatively correlated, while latitude and zooplankton richness showed positive correlations with bacterial richness (Table 2). The three variables jointly explained 15% of the variation in bacterioplankton richness.

**Table 2:** The results of General Linear Model for the zooplankton, phytoplankton, and bacterioplankton richness for the whole set of lakes (n = 100).

	Variable	N	SS	df	MS	F	p	Constant	SE	R^2^
Zooplankton	Constant		0.45	1	0.45	0.06	0.80	0.44	1.78	
	Bacterioplankton richness	100	40.92	1	40.92	5.57	0.02	0.15	0.06	
	Temperature	100	74.42	1	74.42	10.13	0.002	0.27	0.09	
	Phytoplankton richness	100	49.55	1	49.55	6.74	0.01	0.09	0.04	
	Full Model		188.47	3	62.82	8.55	<0.001			0.21
Phytoplankton	Constant		464.58	1	464.58	14.30	<0.001	−97.91	25.89	
	Electricity	100	154.82	1	154.82	4.77	0.032	0.04	0.02	
	Latitude	100	1837.11	1	1837.11	56.55	<0.001	−0.07	0.01	
	Longitude	100	1756.71	1	1756.71	54.07	<0.001	0.17	0.01	
	Total N	100	542.74	1	542.74	16.71	<0.001	0.01	0.02	
	Zooplankton richness	100	301.37	1	301.37	9.28	<0.001	0.61	0.20	
	Full Model		2848.85	5	569.77	17.54	<0.001			0.48
Bacterioplankton	Constant	100	59.29	1	59.29	3.53	0.06	32.63	17.36	
	Latitude	100	141.90	1	141.90	8.46	0.005	0.02	0.01	
	Longitude	100	135.49	1	135.49	8.07	0.005	−0.05	0.02	
	Zooplankton richness	100	118.05	1	118.05	7.03	0.009	0.38	0.14	
	Full Model	100	285.33	3	95.11	5.67	0.001			0.15

The best models were identified with Akaike's Information Criterion (AIC).

Finally, we conducted separate correlation analyses to test the cross-taxon concordance between the three organism groups. We found that zooplankton richness was significantly correlated with both phytoplankton and bacterioplankton richness (R = 0.231; P = 0.019 and R = 0.274; P = 0.006, respectively) (Figure S3). However, phytoplankton and bacterioplankton richness were not correlated (R = 0.022, P = n.s.).

The taxonomic details of the community compositions observed in this study can be found in [38].

## Discussion

Lakes are largely underutilized, but highly useful for studying the PDR, as they are bounded ecosystems embedded in a terrestrial matrix and enumeration of locally coexisting species is thus relatively reliable. For example, earlier works by Dodson et al [39], Hessen et al [40] and Ptacnik et al [24] have shown that there may be predictable large-scale patterns in plankton richness mediated by productivity. The PDR is often studied using small-scale field experiments, or studies are conducted in laboratory microcosms [11], [41], [42]. However, species diversity may have an even higher effect on productivity in natural unmanipulated systems than in artificial ecosystems [43]. The caveats for experimental studies can include small spatial scale, small temporal extent of the study, the lack of natural disturbances and unnatural species compositions that do not exist in nature [28], [44]–[46]. Therefore, large-scale observational studies are also fundamental to long-term biodiversity inventories and conservation programs [47]. Our study is, to our knowledge, the first study that examines PDR at large scales and that also considers microbial organisms including lake bacteria.

Regardless of the great potential of lakes for studying PDR, our survey showed large variability in the PDR among the five drainage systems for all planktonic groups. We initially predicted that the relationship between species richness and productivity would be unimodal at a small scale and positive linear at a larger scale (Fig. 1a, [19], [20]). At the small scale, the observed relationships varied, however, from non-significant and negative linear to significant positive linear and unimodal [see also 25], [48]. This is in line with Witman et al [48] who also found variable PDRs in Arctic macrobenthos indicating that PDRs may often be highly context dependent.

At the larger scale instead, we found that two out of three PDRs were significant and positive linear as we expected. Therefore, our hypothesis on scale-dependency in PDR was partly supported. The reason for the lack of clear relationships within drainage-systems remain speculative at present but may be related to facts that (i) planktonic organisms were overall largely driven by some other factors than productivity and (ii) productivity gradients were not long enough for producing a possible “hump-shaped” PDR in these unmanipulated systems. However, we would like to emphasize that the study by Chase & Leibold [20] was conducted in much smaller spatial extent than our study as they compared PDR within single pond with PDR among multiple ponds sampled in one drainage system only (versus our 20 lakes sampled in five drainage systems). They collected samples twice per year, over two years. Therefore, their findings are not fully comparable to our results because of substantially larger spatial scale in the present study and different amounts of sampling occasions.

Besides studying the scale-dependency of the PDR, one of our main goals was to investigate if increasing species richness is related to higher levels of phytoplankton biomass. Traditionally, biomass is expected to be driven by nutrient availability (e.g. [49]). However, recent studies have viewed the PDR from a different angle, asking how species richness can control biomass production instead of only responding to it [8]. We found that species-rich communities also maintained higher biomass than communities consisting of fewer species. Our results thus seem to agree with Ptacnik et al. [50] who found that resource use efficiency (RUE, calculated as a ratio between chl a and total P) and phytoplankton richness were positively correlated. We would like to emphasize, though, that in our data, phytoplankton biomass was slightly more strongly related to community composition summarized by the NMDS 1 scores of sites than to the pure species number at each site. This may indicate that composition effects are nonetheless stronger than pure richness effects in our study system. This is in line with Downing & Leibold [28] who observed that species composition within richness levels can have equal or more marked effects on functions than average effects of richness in pond ecosystems. We admit, though, that teasing apart the richness effect from the composition effect in our study is not clear-cut because of field observations only - one would need carefully replicated experiments in the field to examine this more closely. For example, it is likely that the composition effect is mediated by changes in resource availability, and resource availability seems strongly affect the amount of biomass in our study system (Fig. 5).

As multiple ecosystem processes may act simultaneously, we studied the concomitant pathways between richness, resource availability, resource ratio and biomass for the phytoplankton communities and formally tested the multivariate hypothesis of the PDR introduced by Cardinale et al [8]. The results of the SEM analysis showed a strong pathway between resource availability and biomass, thus agreeing with the results of Cardinale et al. [8]. It should be noted, however, that biomass is not determined by resource availability only, as there was a positive link between richness and biomass. This finding suggests that richness is related to more efficient ecosystem production. Cardinale et al [8] also proposed that as resources become increasingly imbalanced, biomass production slows down. However, we could not detect such a negative effect of resource imbalance on standing biomass. Rather, our data showed a positive, albeit relatively weak effect of resource ratio on biomass. Moreover, we did not find a strong pathway between resource availability and species richness. This counterintuitive result is in line with e.g. Longmuir et al [30], and Dodson et al [39] who did not detect clear relationships between resource availability and species richness. Altogether, we could explain quite reasonable proportion (32%) of phytoplankton biomass using resource availability, resource ratio and phytoplankton richness alone and thus conclude that our data partly support the multivariate hypothesis by Cardinale et al [8].

As productivity alone could nonetheless explain only a relatively small portion of the variability in species richness for all three planktonic groups, we studied whether species richness was correlated with some other factors. In general, it seemed that factors related to productivity were not often incorporated into the best regression models. For zooplankton, water temperature was positively correlated with species richness. We speculate that the positive relationship between richness and temperature may stem from higher energy-input supporting more species as predicted of the species-energy theory [2], [51]. Zooplankton results further suggest that there is concordance in richness between different trophic levels as both phytoplankton and bacterioplankton richness were included in the best GLM model for zooplankton. As bacterioplankton and zooplankton richness were positively related, this means that the positive feedbacks between trophic levels can maintain species diversity in these communities. Positive correlations in richness between the trophic levels have been found in several studies of terrestrial systems [52]–[54], but in aquatic ecosystems correlations in richness across trophic levels have been weak or non-significant [30], [55], [56]. Due to these disparate results, it has been suggested that the degree of concordance in species richness patterns among trophic levels generally differ between terrestrial and aquatic systems [30]. In our study, the major environmental factors affecting species richness were different for each trophic level. One may suggest that that the similar accumulation of species across trophic levels may be driven by species interactions between trophic levels in the planktonic food web rather than similar responses to environmental gradients. However, the possible cross-taxon concordance remains speculative as we did not study but the lowest levels of food web of the lakes.

As our study organisms ranged from unicellular bacteria to visible meiofauna, we initially expected notable differences in richness patterns between the organism groups. Traditionally, microscopic organisms are expected to be unlimited in their dispersal ability due to their small size and high abundance (e.g. [27]) and thus lack any notable biogeographical patterns. Cross-taxon studies that include bacteria and examine large-scale patterns in biodiversity are still very rare. Our data seem to disagree with the theory of ubiquity of microorganisms, as both phytoplankton and bacterioplankton richness were significantly related to geographical location of the lakes, and surprisingly, zooplankton richness seemed to be the most weakly related to sampling location. Although the location of a lake always includes a signal of unmeasured environmental variables, our results seem to agree with Hillebrand et al [57], Soininen et al [58], and Heino et al [59] on microorganisms having restricted biogeographical distributions perhaps similar to the patterns observed for macroorganisms.

Although we could explain a considerable portion of the variation in species richness using multiple abiotic and biotic factors, some important aspects concerning the PDR remain speculative. For instance, we were not able to estimate the importance of colonization or extinction on patterns in the PDR in our study regions due to static snapshot sampling for each site. We were also unable to assess fish diversity and abundance in the lakes, although predation can reduce interspecific competition and thus promote species coexistence of the zooplankton, for example [40]. Further, other vital factors, such as disturbances [60]–[62], chemical and thermal variability [63], evolutionary history [64], or the history of community assembly [65], can also influence the patterns in species richness. The importance of different mechanisms behind the PDR (e.g. sampling effect and complementarity) also remain equivocal as the independent effects of these factors cannot be reliably identified using a large-scale field data only. We thus encourage ecologists to further study these factors more thoroughly in aquatic environments.

To conclude, we found that the PDRs are variable in plankton communities of small boreal lakes. These data showed unimodal and linear PDRs at local scale, yet also including many non-significant PDRs. At the regional scale in turn, we found linear PDRs for phyto- and zooplankton and conclude thus that PDR may vary with spatial scale. Our GLM analyses further suggested that there are correlations in richness across trophic levels in freshwater plankton. The concordance likely results from species interactions between the trophic levels as there were no common responses to measured environmental gradients. Finally, we found that both resource availability and species richness contributed to biomass production in phytoplankton.Our study thus emphasizes the need for conserving diversity in order to maintain ecosystem processes in freshwaters.

## Supporting Information

Table S1 **Environmental variables.** Means and ranges for the main environmental variables for each drainage system.
(TIF)

Table S2 **The results of Structural Equation Modeling.** The individual pathways in the model with (A.) and without (B.) best model selection. The significance is indicated by the P value. Resource availability and resource ratio were significantly related to biomass.
(TIF)

Figure S1 **Accumulation curves.** Species accumulation curves for a–b) zooplankton, c–d) phytoplankton, e–f) bacterioplankton data sets. The left column indicates the accumulation of species in the most species-rich areas and the right column shows the accumulation curves in the most species-poor areas.
(TIF)

Figure S2 **The relationships between species richness and total P.** The relationships between local species richness and total P (µg/l) in zooplankton (a–e), phytoplankton (f–j), and bacterioplankton (k–o) for data sets at five drainage systems each consisting of 20 lakes. Solid lines indicate significant relationships between species richness and total P. Dashed lines denote non-significant relationships. Linear or quadratic model was used depending on the AIC value (see Table 1).
(TIF)

Figure S3 **Cross-taxon concordance.** Concordance between observed richness for a) zooplankton and phytoplankton (P = 0.019), b) bacterioplankton and phytoplankton (P = n.s.), and c) bacterioplankton and zooplankton (P = 0.006).
(TIF)
