# Supplementary figures and images for: Productivity-Diversity Relationships in Lake Plankton Communities
Source: PLoS One. 2011 Aug 5;6(8):e22041. doi: 10.1371/journal.pone.0022041 (PMC3151241; doi:10.1371/journal.pone.0022041)

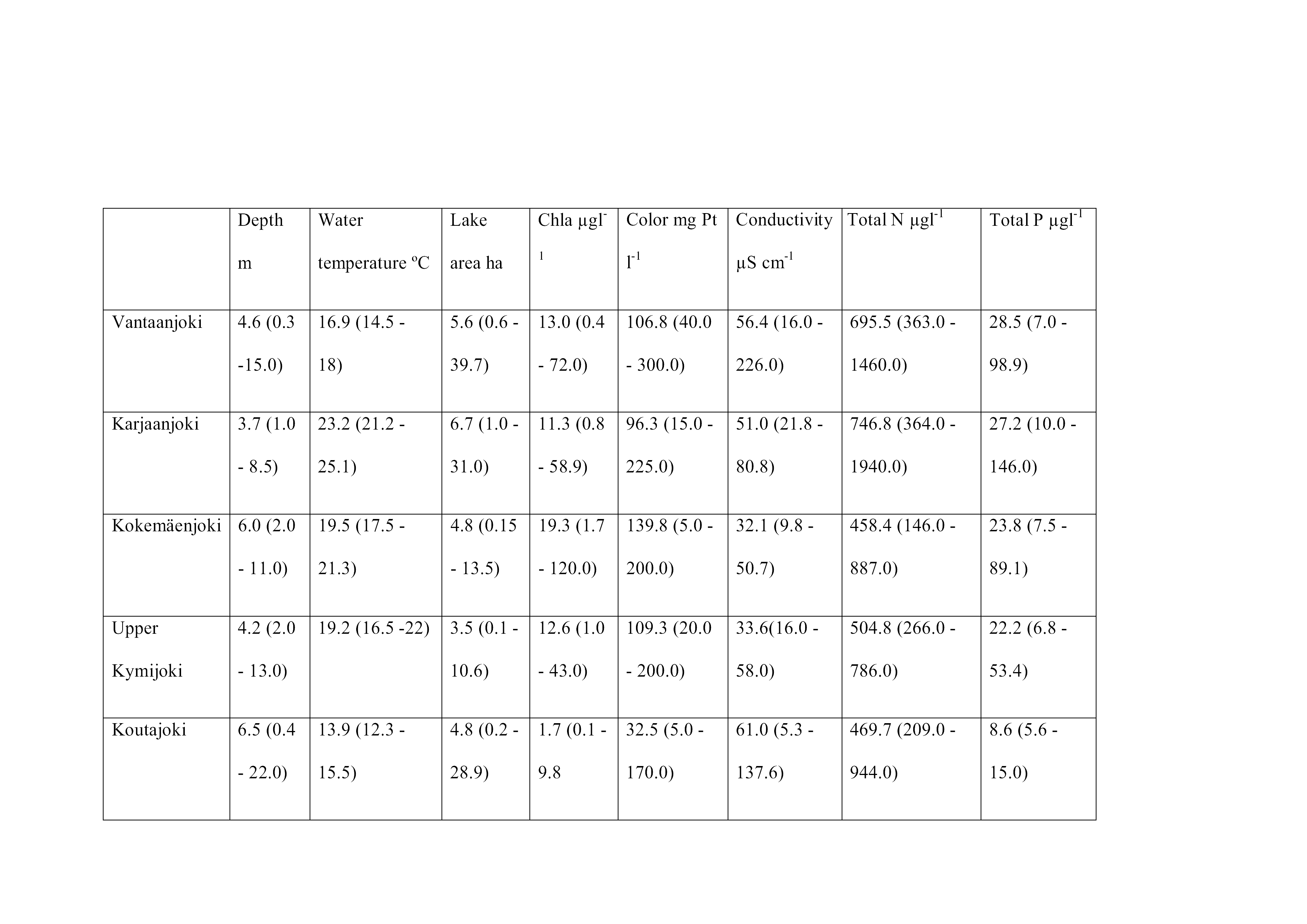

Supplement: Table S1 — Environmental variables. Means and ranges for the main environmental variables for each drainage system. (TIF) [file pone.0022041.s001.tif]

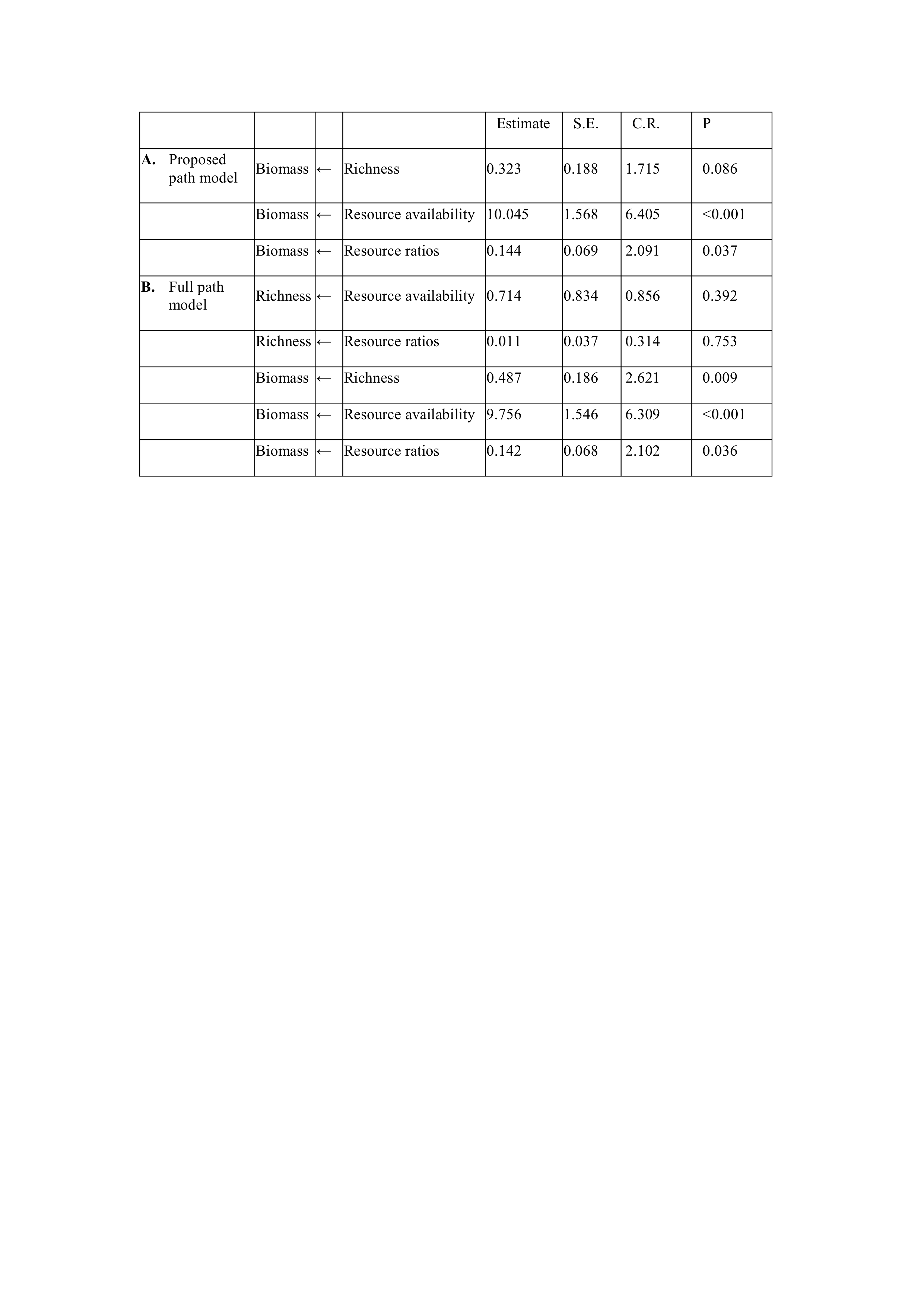

Supplement: Table S2 — The results of Structural Equation Modeling. The individual pathways in the model with (A.) and without (B.) best model selection. The significance is indicated by the P value. Resource availability and resource ratio were significantly related to biomass. (TIF) [file pone.0022041.s002.tif]

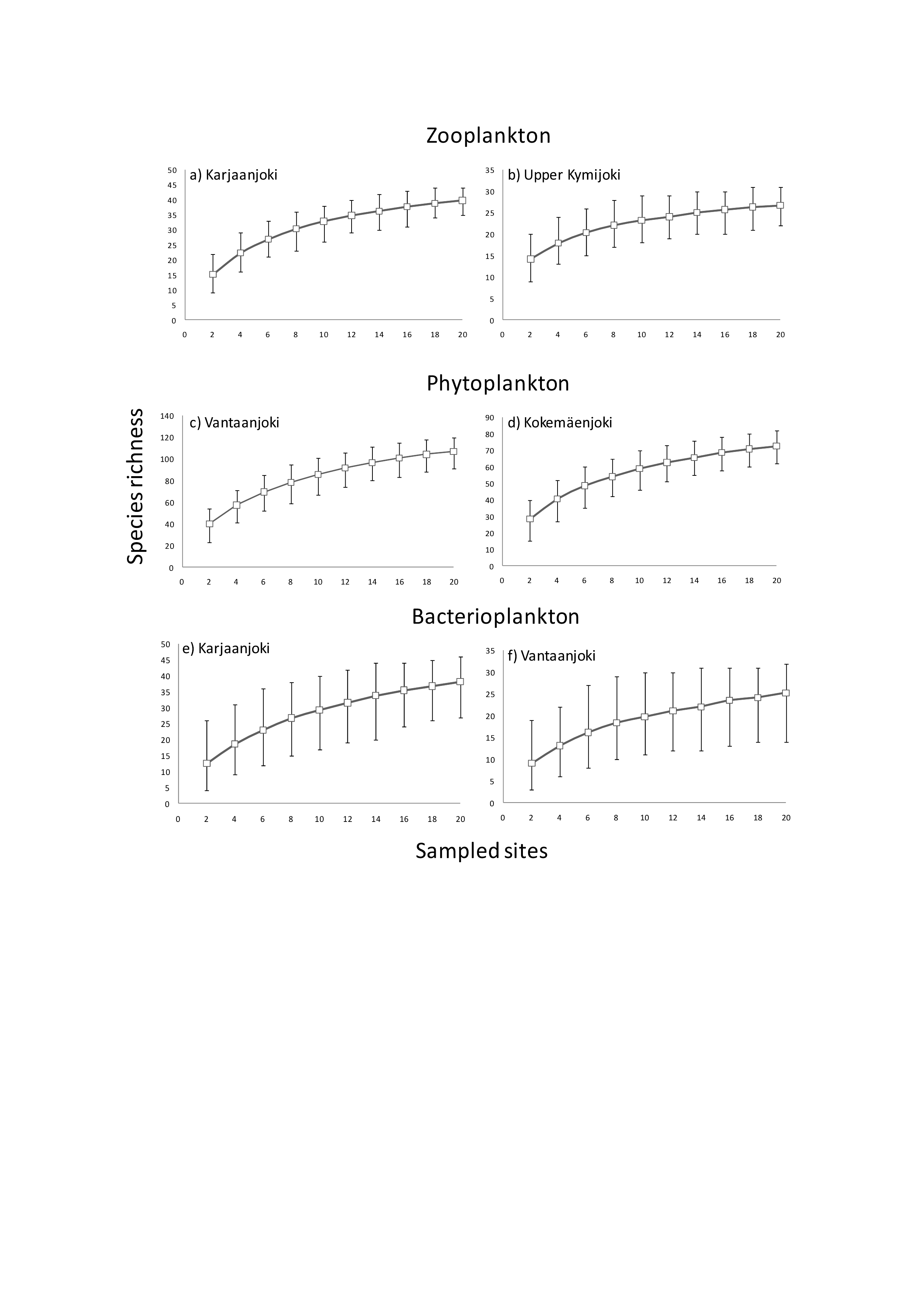

Supplement: Figure S1 — Accumulation curves. Species accumulation curves for a–b) zooplankton, c–d) phytoplankton, e–f) bacterioplankton data sets. The left column indicates the accumulation of species in the most species-rich areas and the right column shows the accumulation curves in the most species-poor areas. (TIF) [file pone.0022041.s003.tif]

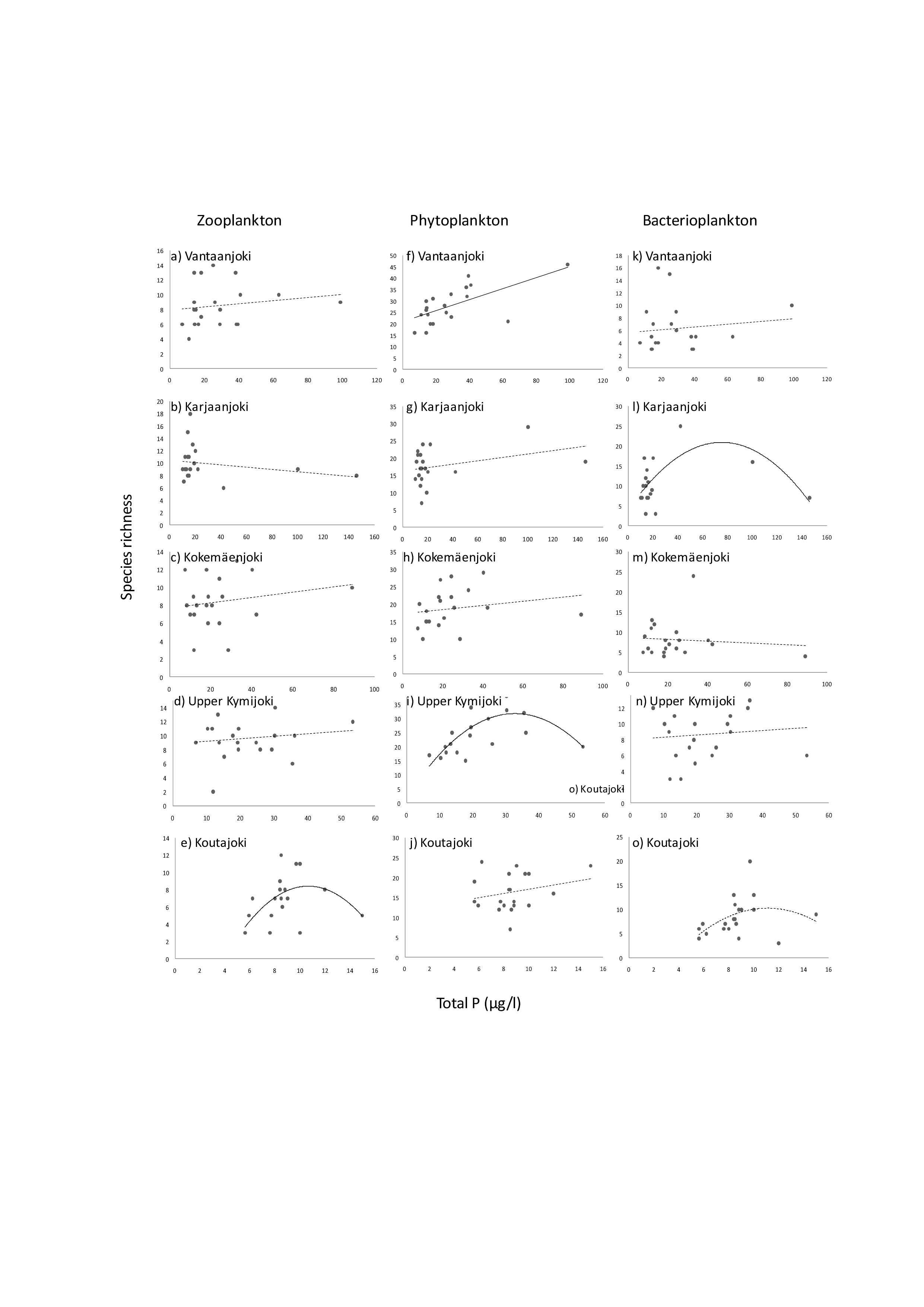

Supplement: Figure S2 — The relationships between species richness and total P. The relationships between local species richness and total P (µg/l) in zooplankton (a–e), phytoplankton (f–j), and bacterioplankton (k–o) for data sets at five drainage systems each consisting of 20 lakes. Solid lines indicate significant relationships between species richness and total P. Dashed lines denote non-significant relationships. Linear or quadratic model was used depending on the AIC value (see Table 1). (TIF) [file pone.0022041.s004.tif]

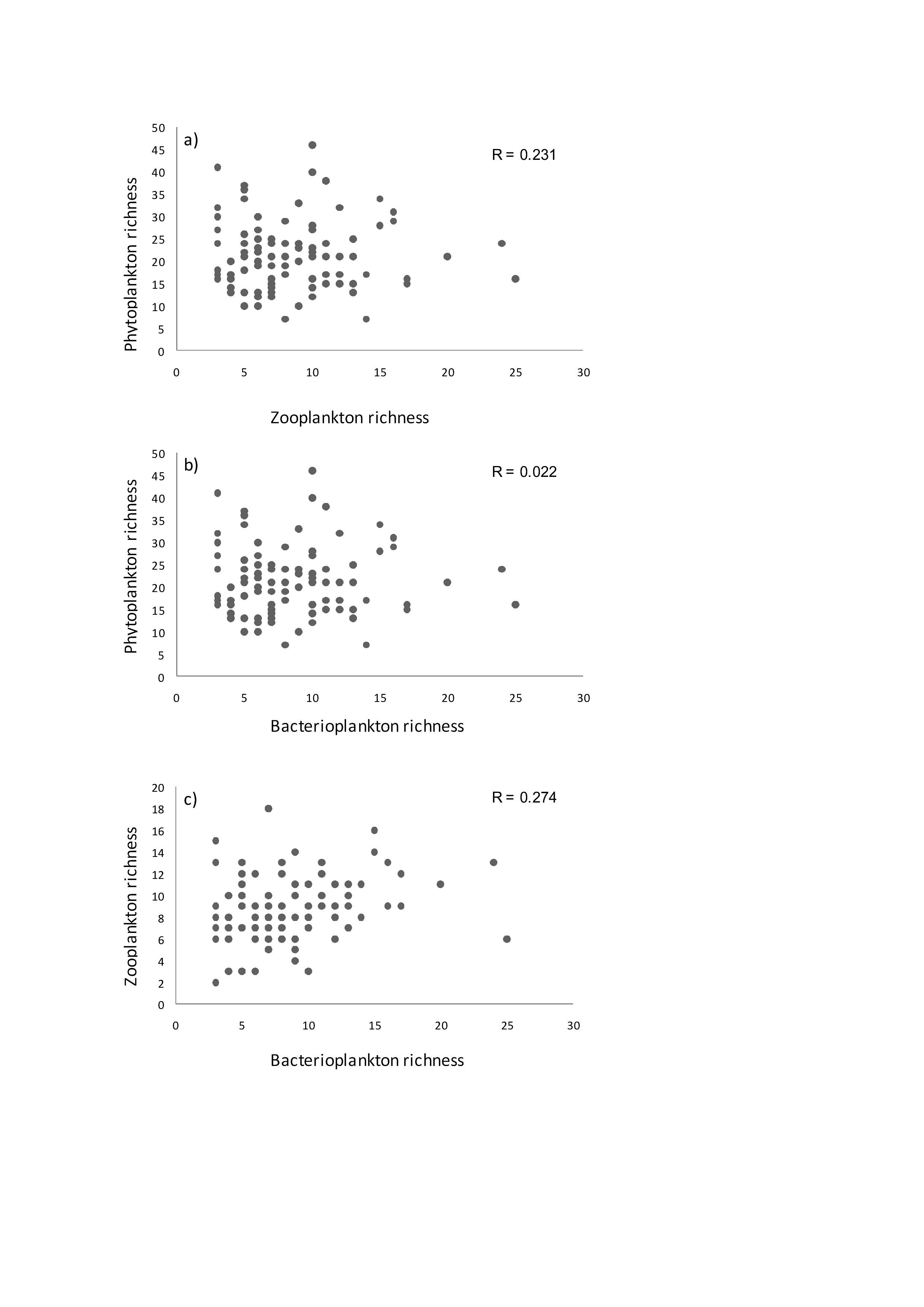

Supplement: Figure S3 — Cross-taxon concordance. Concordance between observed richness for a) zooplankton and phytoplankton (P = 0.019), b) bacterioplankton and phytoplankton (P = n.s.), and c) bacterioplankton and zooplankton (P = 0.006). (TIF) [file pone.0022041.s005.tif]
